# Self-Esteem in Hearing-Impaired Children: The Influence of Communication, Education, and Audiological Characteristics

**DOI:** 10.1371/journal.pone.0094521

**Published:** 2014-04-10

**Authors:** Stephanie C. P. M. Theunissen, Carolien Rieffe, Anouk P. Netten, Jeroen J. Briaire, Wim Soede, Maartje Kouwenberg, Johan H. M. Frijns

**Affiliations:** 1 Department of Otorhinolaryngology and Head & Neck Surgery, Leiden University Medical Center, Leiden, The Netherlands; 2 Department of Developmental Psychology, Leiden University, Leiden, The Netherlands; 3 Dutch Foundation for the Deaf and Hard of Hearing Child, Amsterdam, The Netherlands; 4 Leiden Institute for Brain and Cognition, Leiden, The Netherlands; McGill University, Canada

## Abstract

**Objective:**

Sufficient self-esteem is extremely important for psychosocial functioning. It is hypothesized that hearing-impaired (HI) children have lower levels of self-esteem, because, among other things, they frequently experience lower language and communication skills. Therefore, the aim of this study was to compare HI children's self-esteem across different domains with those of normal hearing (NH) children and to investigate the influence of communication, type of education, and audiological characteristics.

**Methods:**

This large (N = 252) retrospective, multicenter study consisted of two age- and gender-matched groups: 123 HI children and 129 NH controls (mean age  = 11.8 years). Self-reports were used to measure self-esteem across four domains: perceived social acceptance by peers, perceived parental attention, perceived physical appearance, and global self-esteem.

**Results:**

HI children experienced lower levels of self-esteem regarding peers and parents than NH controls. Particularly HI children who attended special education for the deaf were at risk, even after correcting for their language development and intelligence. Yet, levels of global self-esteem and self-esteem involving physical appearance in HI children equalled those of NH controls. Furthermore, younger age at implantation and longer duration of having cochlear implants (CIs) were related to higher levels of self-esteem.

**Conclusion:**

HI children experience lower levels of self-esteem in the social domains. Yet, due to the heterogeneity of the HI population, there is high variability in levels of self-esteem.

**Discussion:**

Clinicians must always be aware of the risk and protective factors related to self-esteem in order to help individual patients reach their full potential.

## Introduction

Self-esteem refers to one's general evaluation or appraisal of the self, including feelings of self-worth [1]. Besides an evaluation of the self, self-esteem also denotes how one values oneself. This basic appreciation of the self has effects on multiple dimensions in our lives, such as our friendships, our successes, and our academic career. Moreover, individuals with higher levels of self-esteem are better able to cope with stressful life events [1], whereas lower levels of self-esteem are associated with more loneliness, peer rejection, aggression, delinquency, and psychopathology [2]–[6]. Hence, it is of the utmost importance to have a sufficient level of self-esteem.

One would assume that hearing-impaired (HI) individuals encounter more difficulties regarding their self-esteem because they often face multiple challenges, such as speech and language delays, communication problems, and less or no access to the sound-dominated world [7]. These problems could potentially harm HI children's level of self-esteem, resulting in for example less stable friendships and more bullying [8]. Well-developed language and communication skills have been linked to higher levels of self-esteem [9]. Nowadays, deaf children who can have no or minimal benefit from conventional hearing aids receive cochlear implants (CIs), which considerably change and often improved outcomes for them in the aforementioned domains [10], [11]. Recently, CI recipients have been found to have levels of self-esteem that equal those of NH children [12], [13], which emphasizes the importance of adequate language development for self-esteem.

Studies that looked at levels of self-esteem in a more heterogeneous group of HI children showed inconsistent results. When compared to normal hearing (NH) peers, some researchers reported lower self-esteem in children with mild to profound hearing losses [14]–[16], while others demonstrated that levels of self-esteem were similar to those of NH counterparts [12], [13], [17]–[19]. In the literature, no consensus has been reached for the effect of type of education on HI children's self-esteem: some researchers showed higher self-esteem in HI children attending mainstream education than the ones attending special education, whereas others found no difference [19]–[21]. Possibly, HI children evaluate their abilities differently in different school contexts. Whilst HI children attending special schools evaluate themselves within a compatible peer group, HI children in a mainstream setting will compare themselves with their hearing peers. [21]. Conversely, it could also be argued that HI children attending mainstream schools actually feel a higher self-worth, because they are able to fit in with hearing peers, which can be perceived as a major achievement.

Self-esteem is often conceptualized as being multidimensional, consisting of several specific domains that are related to various facets of life (e.g. perceived parental attention, social acceptance by peers and physical appearance), as well as a more general view of oneself, often called ‘global self-esteem’ [3], [22]. Levels of self-esteem can vary considerably across these different domains, particularly during adolescence, as this is a transition phase marked by crucial emotional and behavioral changes [3], [23]. Parents become less influential, while close friends' and classmates' judgments become increasingly important [24]. Attention to and perception of one's physical appearance also increase. A child may be at risk of low self-esteem in one specific domain but not in another [21]. Although it has been postulated that self-esteem interventions do not directly improve outcomes, being aware of these distinctions can support the caregiver when helping or counseling the child [2].

Besides the contrasting findings of past research regarding differences in HI and NH children's levels of global self-esteem, there is a paucity of data concerning the more specific domains of self-esteem in HI children compared to NH children. Only a few studies have reported on specific domains of self-esteem in HI children when compared to NH controls. These found that the HI children had more difficulties regarding peer acceptance and family relations although they felt equally confident about their physical appearance [15], [19], [25]. To the best of our knowledge, no other studies have been performed to date in which these specific domains were studied and compared in both HI and NH children.

Hence, our goal here was not only to investigate the level of global self-esteem in a large and diverse sample of HI and NH children and adolescents, but also to examine three more specific domains of self-esteem: perceived social acceptance by peers, perceived parental attention and perceived physical appearance. Secondly, we wanted to study whether language development and communication skills, type of education, and audiological characteristics would influence the level of self-esteem. Based on (the majority of) the existing literature, we expected that adequate communication skills would result in higher self-esteem [9], [12], [13] and that children attending special education would have lower self-esteem than children in mainstream education [19]–[21]. Concerning audiological factors, no recent studies were available on which to base our predictions. Therefore, we have performed several explorative analyses to see whether relations between these factors and the different domains of self-esteem exist.

## Materials and Methods

### Participants

A total of 252 children (Mean age  = 11.8 years, *SD* = 1.7) participated in this study of which 123 were HI children and 129 were NH controls. All children had a nonverbal IQ of at least 80, and no other known learning problems. Children were not included if they experienced comorbidities such as visual impairment or Autism Spectrum Disorders. The HI children were included if they experienced a loss of at least 40 decibels in the best ear, which was detected prelingually (<3 years) or perilingually (3–5 years). Table 1 shows the characteristics of all included children. For the CI recipients specifically, the mean age at implantation was 3.8 years (*SD* = 2.7; range  = 0.9–10.8 years). The mean duration of CI use was 8.3 years (*SD* = 2.6; range 0.8–13.0 years). Most CI users (*n* = 40; 76%) had one CI, and 13 (24%) children were bilaterally implanted.

**Table 1:** Characteristics of all participants.

	Total sample (N = 252)		HI sample (*n* = 123)	
	Controls	HI	CI	Hearing aid
Number of children – *n*	129	123	53	70
Age mean in years (*SD*)	11.6 (1.3)	12.0 (1.8)	11.9 (2.1)	12.0 (1.7)
Gender - *n* (%)				
Male	58 (45%)	60 (49%)	24 (45%)	36 (51%)
Female	71 (55%)	63 (51%)	29 (55%)	34 (49%)
Socioeconomic status mean (*SD*)^a^	12.1 (2.4)	11.5 (2.3)	11.7 (2.3)	11.3 (2.4)
Nonverbal IQ				
IQ norm score Picture arrangement (*SD*)	10.6 (3.4)	10.2 (3.5)	9.9 (3.5)	10.4 (3.5)
IQ norm score Block design (*SD*)	10.6 (3.0)	10.4 (3.1)	10.3 (2.8)	10.5 (3.4)
Spoken language skills^b^				
Sentence comprehension (*SD*)	7.1 (2.3)	6.6 (3.1)	6.6 (3.1)	6.7 (3.1)
Story comprehension (*SD*)	7.0 (2.5)	6.3 (2.8)	5.6 (3.0)	6.8 (2.6)
Sign language skills^c^				
Sentence comprehension (*SD*)	-	2.3 (0.9)	2.1 (1.0)	2.3 (1.0)
Story comprehension (*SD*)	-	2.6 (0.7)	2.8 (0.8)	2.5 (0.7)
Children's Communication Checklist^d^				
General Communication Composite (*SD*)	73.9 (18.2)	91.3 (18.2)^1^	91.9 (18.4)	90.8 (18.2)
Pragmatic Composite (*SD*)	36.2 (9.1)	46.6 (8.7)^1^	47.3 (9.1)	46.1 (8.5)
*Audiological variables*				
Degree of hearing loss - *n* (%)^e^				
Moderate (40–60 dB)	-	29 (24%)	0 (0%)	29 (41%)^1^
Severe (61–90 dB)	-	25 (20%)	1 (2%)	24 (34%)^1^
Profound (>90 dB)	-	61 (50%)	50 (94%)	11 (16%)^1^
Unknown	-	8 (6%)	2 (4%)	6 (9%)
Preferred mode of communication - *n* (%)				
Oral language only	-	88 (71%)	36 (68%)	52 (74%)
Sign-supported language	-	33 (27%)	17 (32%)	16 (23%)
Sign language only	-	2 (2%)	0 (0%)	2 (3%)
Type of education – *n* (%)				
Regular education	-	74 (60%)	32 (60%)	42 (60%)
Special education	-	49 (40%)	21 (40%)	28 (40%)
Mean age at onset in years (*SD*)	-	1.6 (1.3)	1.2 (0.9)	1.9 (1.5)^2^
Age at onset of hearing loss - *n* (%)				
Prelingual (<3 yrs)	-	104 (85%)	49 (93%)	55 (79%)^3^
Perilingual (3–5 yrs)	-	12 (10%)	2 (4%)	10 (14%)^3^
Unknown	-	7 (6%)	2 (4%)	5 (7%)
Mean age at 1^st^ hearing aid in years (*SD*)	-	2.1 (1.4)	1.5 (0.9)	2.6 (1.5)^1^

a Socioeconomic status score was measured by parental education, job, and net income. (Unfortunately, due to privacy reasons, almost half of the parents did not fill out the question concerning the net income, so these were not taken into account.)

b Spoken language skills were derived from the Clinical Evaluation of Language Fundamentals; see Materials section for more information.

c Sign language skills were derived from the Assessment Instrument for Sign Language of the Netherlands; see Materials section for more information.

d Higher scores indicate more (social) language problems. More than 70% of the parents responded.

e Degree of hearing loss was calculated by averaging unaided hearing thresholds at 500, 1,000, and 2,000 Hertz.

1 p<.001;

2 p<.01;

3 p<.05.

### Procedure

The NH controls were recruited from primary and secondary mainstream schools across the Netherlands to reach a geographically and socio-economically diverse sample. To collect a sample that represented the complete spectrum of HI children, we recruited from 14 (both primary and secondary) mainstream schools and special schools for the HI (schools that supported development of auditory and oral skills, with or without the use of signs), 2 hospitals, 5 Speech and Hearing centers or residential schools, and via newsletters in the Netherlands and the Dutch-speaking part of Belgium.

The questionnaire was administered on a laptop. Questions appeared one by one on the screen. Instructions for all tests were provided in the child's preferred mode of communication to ensure that the child understood. The HI children could choose between two versions of the questionnaire: the first version which comprised written items exclusively, and the second version in which each item was presented in written text and sign language simultaneously by means of a video clip in the upper right-hand corner of the screen. Translation from spoken language into sign language was performed by a qualified interpreter and back translation of all signed items showed good convergence with the original items.

Parents or caregivers were requested to complete a questionnaire assessing demographic variables such as net income and level of education. In the HI group, several audiological variables were derived from the child's medical and audiological notes after informed consent was given. SES was calculated as the mean of parental education, job, and net income. Unfortunately, due to privacy reasons, almost half of the parents did not fill out the question concerning net income, so these were not taken into account.

### Ethics statement and privacy regulation

Approval for the study was obtained by the Medical Ethics Committee of the Leiden University Medical Center under number P10.137, and carried out in accordance with the standards set out by the Declaration of Helsinki. All parents or caregivers gave written consent for their child's participation prior to data collection. Next to parents and caregivers, all children aged 12 or older gave written consent as well. Before the assessment started, all children were assured that their responses would be processed anonymously.

### Self-esteem questionnaire

To assess self-esteem, the self-report *Children's Self-Confidence and Acceptance Scale* [26], [27] was used, which had only been used in NH children previously. The scale showed a strong convergent validity with the CBSK, which is the well-established Dutch version of Harter's self-esteem scale (*The Self-Perception Profile for Children* [28], [29]). Harter's scale was used because we wanted to address the different specific domains of self-esteem instead of the more general global self-esteem measured by the Rosenberg self-esteem scale [30]. The items of the questionnaire were formulated by a team of child psychologists, targeting key aspects of self-esteem. Sentences were formulated short and simple, so HI children with language comprehension problems would be able to understand these items and respond to them coherently. The reason for choosing a self-report instead of parent or teacher reports is that self-reports give the most accurate scores when measuring self-esteem [31], [32].

The questionnaire represents three relevant domain-specific categories, and one overall category, that could be answered on a 3-point Likert scale:

1. The *perceived social acceptance by peers* (‘peers’, 5 items) domain examines the perception of the child of how well he or she is accepted by peers or feels popular (Example item: “Children ask to play with me”).
2. The *perceived parental attention* (‘parents’, 7 items) domain assesses the self-perceived degree to which parents or caregivers are interested in and give support to the child's thoughts and needs (“My father or mother are happy with me”).
3. The *perceived physical appearance* (‘physical appearance’, 5 items) domain reflects the child's idea of how good-looking or attractive he or she is (“Other children think my appearance is nice”).
4. The *global self-esteem* (‘global’, 5 items) measures the child's perceptions of general statements concerning the self (“I am happy with myself”). These five items address comparable issues to those used in Rosenberg's self-esteem scale [30].

Children were asked to rate the items on a 3-point Likert-type scale (1 =  *not true*, 2 =  *sometimes true*, 3 =  *often true*). The internal consistency was good for both the HI and the NH group (Table 2).

**Table 2:** Psychometric properties of the four domains of self-esteem.

	Range	Number of items	Inter-item correlation	Cronbach's Alpha	
				HI	NH controls
Domains of self-esteem					
Perceived social acceptance by peers	1–3	5	.75	.74	.75
Perceived parental attention	1–3	7	.34	.76	.75
Perceived physical appearance	1–3	5	.46	.83	.78
Global self-esteem	1–3	5	.25	.66	.60

### Language development and communication skills

Language development and communication skills were measured because of their known positive influence on self-esteem [9]. Two types of language development were assessed: *sentence comprehension* and s*tory comprehension*. HI children using spoken language and NH controls received two corresponding subtests of the Dutch version of the *Clinical Evaluation of Language Fundamentals - Fourth Edition* (CELF) [33], [34]. HI children who use sign or sign-supported language received specific subtests of the *Assessment Instrument for Sign Language of the Netherlands* [35]. All original language scores were transformed to norm scores and these were corrected for chronological age. The sentence comprehension task was not administered to 10 HI and 16 controls and the story comprehension task was not administered to 5 HI and 16 NH controls.

The *Children's Communication Checklist version 2* was used to evaluate communication skills indicated by the parents or caregivers [36]. This questionnaire, consisting of 70 items, has been predominantly designed to assess social and pragmatic language of children aged 4 to 16, although it also assesses other qualitative aspects of language. The checklist contains eight scales: speech production, syntax, semantics, coherence, inappropriate initiation, stereotyped conversation, use of context, and non-verbal communication. Two composite scores are conventionally obtained from these scales: the general communication composite (GCC) and the pragmatic composite (PC). Each item can be scored from 0 (*never or less than once a week*) to 3 (*several times a day or always*). Higher scores indicate more (social) language problems. To the parents of the HI children using sign or sign-supported language, the speech production and syntax scales were not administered.

### Intelligence

An index of the nonverbal intelligence was obtained with two tests from the *Wechsler Intelligence Scale for Children - Third Edition*: block design by copying geometric designs with cubes, and picture arrangement by sequencing pictures to make logical stories [37], [38]. All raw scores were converted into age-equivalent norm scores based on Dutch standards (10 =  average). A random sampling (*n* = 23) across HI children who were previously assessed with a complete intelligence test (either the Snijders-Oomen nonverbal intelligence test [39] or the WISC) showed a high correlation between the scores of our tests and the IQ score, *r* = .79, *p*<.001. The tasks were not administered to 8 HI and 17 NH children, due to time constraints.

### Statistical analyses

First, in order to compare the levels of the specific domains of self-esteem between HI and NH children, Multivariate Analysis of Variance (MANOVA) and Multivariate Analysis of Covariance (MANCOVA) were used. In the MANCOVAs, several covariates were incorporated one by one, including intelligence, socio-economic status (SES) and language and communication skills. For the second and third research questions (i.e., influence of communication skills and type of education on the different domains of self-esteem, respectively) MANCOVAs were performed, and confounding variables were included one by one in case of group differences. Several continuous audiological factors (e.g., duration of CI use, age at implantation) and their association with the different domains of self-esteem were addressed by Pearson's correlations. Nominal variables (uni- or bilateral CI, pre-or perilingual onset of HI) were compared by means of MANOVAs. When a score or variable was not available, the participant was excluded from the analysis concerned. It was checked whether there were group differences on age, gender, SES, and type of hearing device between those who completed and those who did not complete all the questionnaires and this was not the case. The program *Statistical Packages for the Social Sciences* (version 20.0) was used.

## Results

### Self-esteem in HI versus NH children

**Figure 1:**
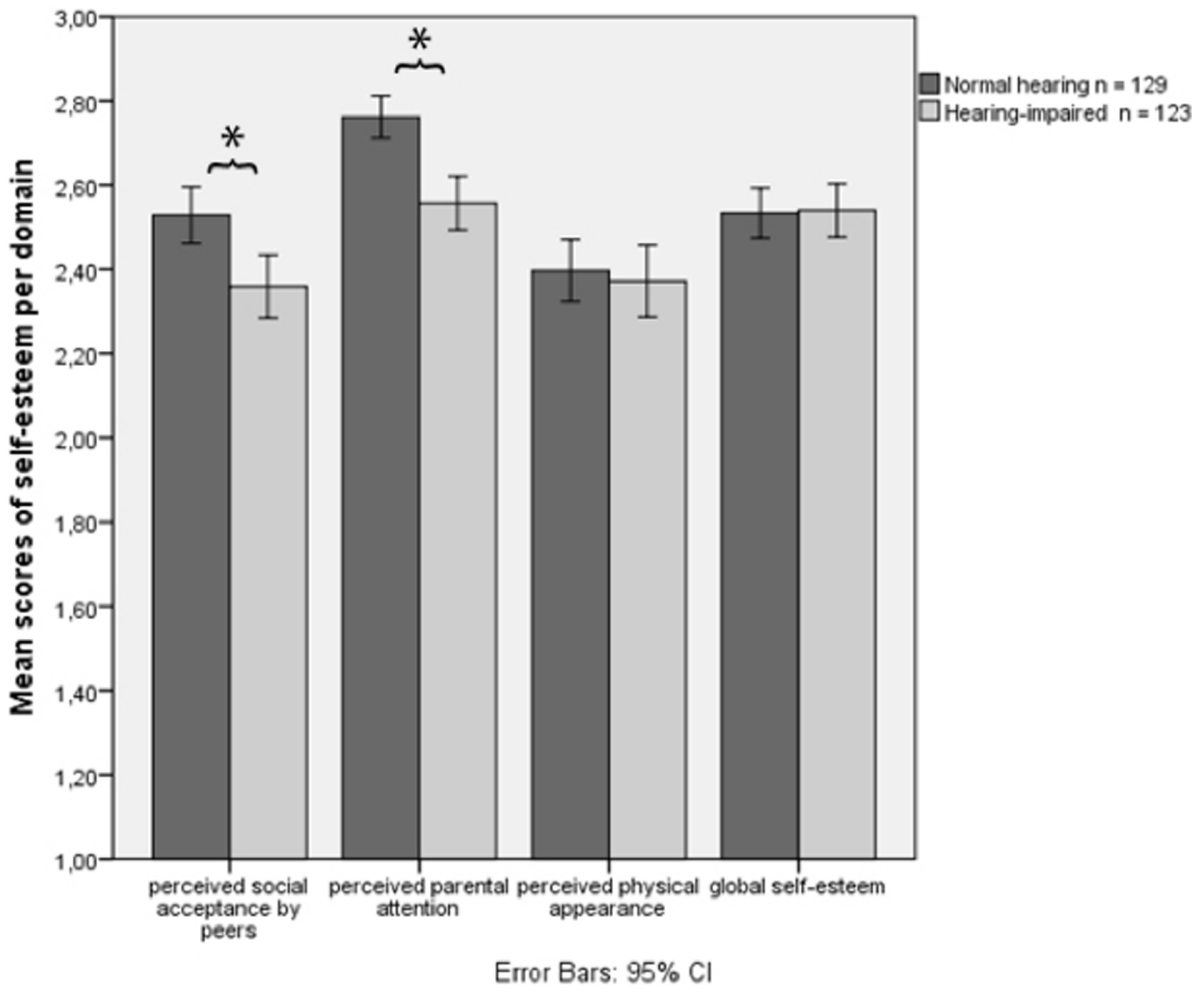
Mean scores of self-esteem per domain. *p<.05.

Regarding global self-esteem, the scores of NH and HI children did not significantly differ (Δ = −.007, *p* = .881). To compare the groups with respect to their specific domains of self-esteem, a MANOVA was carried out with group (NH or HI) as the between-subjects variable and the levels of self-esteem in each of the specific domains as the within-subjects variable. This analysis revealed a main effect for self-esteem *F_HF_*(1.62, 403.73) = 56.78, *p*<.001 *η_p_^2^* = .19, and for group *F*(1, 250) = 11.77, *p* = .001 *η_p_^2^* = .05 which was qualified by a group x self-esteem interaction effect *F_HF_*(1.62, 403.73) = 6.16, *p*<.01 *η_p_^2^* = .02. Post-hoc t-tests showed that HI children had lower self-esteem than NH controls on two domains: the peers' domain (Δ = .20, *p*<.002) and the parents' domain (Δ = .20, *p*<.001) (Figure 1). For the physical appearance domain, no significant group difference was found. A MANCOVA was performed in which we controlled for several important variables (age, gender, intelligence, and SES). The above-described effects retained their significance, so these results were omitted from the results presented here.

When comparing children wearing HAs with those using CIs, the groups did not significantly differ on their level of global self-esteem (Δ = .086, *p* = .18). A 2 (HA or CI) x 3 (domains of self-esteem) MANOVA also revealed no significant differences between the groups in the different domains of self-esteem *F*(1, 121) = .014, *p* = .91 *η_p_^2^*<.001.

### Language development, communication skills and self-esteem

As expected on the basis of past research, *t*-tests revealed that HI children had lower language and communication skills than NH children (story comprehension, Δ = .8, *p*<.038, general communication composite, Δ = 17.4, *p*<.001, and pragmatic composite, Δ = 10.4, *p*<.001, respectively). Therefore, a 2 (group: HI or NH) x 3 (domains of self-esteem) MANCOVA corrected for language development and communication skills was carried out. Again a main effect for group was detected, which was qualified by a group x self-esteem interaction effect: *Wilks' Λ* = .96 *F*(2, 165) = 3.80 *p* = .024 *η_p_^2^* = .044. Post-hoc MANCOVAs showed slightly different results than the MANOVAs: HI children still reported lower self-esteem with respect to the parents' domain *F* (1, 216) = 4.89 *p* = .028, whereas differences in the peers' domain were no longer statistically significant *F* (1, 216) = .03 *p* = .86.

### Type of education and self-esteem

In order to properly examine levels of self-esteem between HI children in special education (for the HI or deaf) and in mainstream education, these two groups were compared on several factors: age, gender, intelligence, SES, and language and communication skills. HI children attending mainstream education had significantly better language skills (Δ = 3.17, *p*<.001), higher intelligence scores (Δ = 2.22, *p*<.001), and higher communication skills (Δ = −22.69, *p*<.001) than children attending special education.

Regarding global self-esteem, the scores of HI children attending mainstream education did not significantly differ from those attending special education (Δ = .10, *p* = .14). A 2 (type of school: special or mainstream) x 3 (domains of self-esteem) MANCOVA which corrected for language development, communication skills and intelligence revealed a significant difference in the parents' domain only, with children in mainstream education scoring higher then children attending special education: *Wilks' Λ* = .82 *F*(3, 67) = 4.87 *p* = .004 *η_p_^2^* = .18.

### Audiological factors

Finally, a series of Pearson's correlations were carried out to see which continuous audiological factors were associated with the specific domains of self-esteem (Table 3). For the peers' and physical appearance domains and for global self-esteem, no significant associations were detected. However, for the parents' domain, younger age at implantation, and consequent longer duration of having CIs, were related to higher self-esteem: *r* (47) = −.359 *p* = .006 and *r* (47) = .376 *p* = .004 respectively. These correlations remained significant when a correction for age and language development was performed, using partial correlation analyses: *r* (41) = −.28 *p* = .035 and *r* (41) = .28 *p* = .034 respectively. To analyze differences within the CI group between two nominal variables (i.e. uni- or bilateral implantation, and pre- or perilingual detection of hearing loss), a MANOVA was carried out for each variable. The independent variables were uni- or bilateral implantation and pre- or perilingual onset of hearing loss, and the dependent variables were the 3 specific domains of self-esteem. No differences between the groups were found: *Wilks' Λ* = 1.0 *F*(3, 49) = .06 *p* = .98 *η_p_^2^* = .004 and *Wilks' Λ* = .94 *F*(3, 112) = 2.37 *p* = .075 *η_p_^2^* = .06 respectively.

**Table 3:** Pearson's correlations between the four domains of self-esteem and associated variables.

	Domains of self-esteem			
	Perceived social acceptance by peers	Perceived parental attention	Perceived physical appearance	Global self-esteem
Age of onset hearing loss	−.02	−.04	.06	−.04
Age at first hearing aid	−.02	.09	.04	−.10
Age at CI implantation	.16	−.36*	.20	−.08
Duration of CI use	−.21	.38**	−.07	−.07

^*^ *p*<.05;

^**^ *p*<.01 (two-tailed).

## Discussion

Self-esteem is a principal prerequisite for healthy psychosocial development and enables children to adjust to stress or burdens [40]. HI children often face demanding situations, so it might be even more important for them to have sufficient levels of self-esteem. By tapping into self-esteem across a number of domains, a differentiated picture of self-esteem was obtained. First, we found that the levels of global self-esteem and perceived physical appearance of HI children did not significantly differ from those of NH controls, despite the former group wearing external amplification devices visible to those around them. This suggests that HI children do not feel more insecure about their looks than other teenagers around this age, which is a positive finding. However, HI children reported lower self-esteem in the domains of perceived social acceptance by peers and perceived parental attention when compared to NH peers. Adequate language development and communication skills can increase self-esteem in the peers' domain, but not in the parental domain.

The fact that HI children reported lower levels of self-esteem than NH children in the social domains indicates that HI children feel less liked and appreciated by parents and peers. This is in line with other studies with HI children [15], [16], [25]. The reasons for lower self-esteem involving parents could be subjective or objective. Children might perceive that their parents spend less time with them, while in fact parents might spend equal time with them as with their NH children. The quality of contact received by NH versus HI children could be different. Parents usually experience more stress and worries raising a HI than a NH child, because they have to adapt to a new situation which necessitates the investment of time, effort, and resources [41]–[44]. For example, an HI child requires frequent hospitals visits and involvement in intensive rehabilitation programs. Chronic parental stress can influence the child's functioning and development in a negative way (e.g., more behavioral problems and impaired psychological functioning) [8], [45]. First of all, parents are a role model for their children. When parents have difficulties coping with stressful events, children will learn and apply these reactions as well. Secondly, more parental stress will also bring about a less positive atmosphere in the home, creating a less optimal environment for healthy development in children. Thirdly, parents might be focused on the impact of the hearing loss and medical site of this, overlooking the child's emotional need for support and guidance. Possibly, parents try hard to support their HI child by speaking slowly, helping with homework, or explaining difficult words [46]. Yet, HI children might interpret this extra attention as if they are failing or falling behind.

On the other hand, language development and communication skills influenced self-esteem in the peers' domain. This means that HI children's self-esteem regarding peers equals that of NH children when their language and communication skills are well-developed. Still, HI children are born into a sound-dominated world, where the focus lies on spoken language, resulting in less satisfactory communication. For example, making friends can be harder for HI children and they are also more neglected and less accepted by NH peers [16], [47]–[50]. The communication barrier between HI and NH children can function as an obstacle for successful interpersonal relationships and may hamper these children in developing solid social networks [51], [52]. This process may pave the way for social isolation and loneliness, with consequences for the child's self-esteem [53], [54]. Hence, by improving language development and communication skills, the HI child might experience better contact with peers, which in turn would likely improve their self-esteem in this domain. In this respect, it has to be mentioned that language development and communication skills did not differ between hearing aided children and CI recipients in our sample. Though the literature often showed that CI recipients have better skills in this regard [10], [55], most of the literature reports on early-implanted children, while our sample is mainly late-implanted. Therefore, we think that the next generation of CI recipients, with better language and communication will, in turn, have higher self-esteem.

Moreover, this research has revealed that children who attend special education for the HI or deaf have lower self-esteem concerning parents when compared to HI children attending mainstream education. Although we have to bear in mind that HI children with good language skills and/or higher intelligence are more easily referred to mainstream education [20], [56]–[58], this study is the first to show that even after correcting for these variables, children in special education still have lower levels of self-esteem. It could be hypothesized that this stems from reasons related to discrimination or stigma. HI children often have to travel far to attend special education, which results in different environments: they have friends at school and different friends at home. Less contact with peers could hinder bonding and attachment, possibly resulting in lower self-esteem [59]. However, longitudinal studies are needed to reproduce these findings, because a cross-sectional study rules out drawing conclusions about causal relations. Additionally, such longitudinal studies must include larger samples in order to examine the influence of parental and friends' hearing statuses on the level of self-esteem.

A limitation of this study was missing data, especially concerning communication, intelligence and language development. It is possible that this missing data did not occur at random. For example, children who read slowly might not have had enough time to complete all the tests. Yet, comparison showed that the group for which information on these measures was missing did not differ from the group with no missing values on important other variables, including age, gender, SES, and the different domains for self-esteem. This seems to strengthen the basis for our conclusions, but future studies are needed to confirm these outcomes.

To conclude, self-esteem in HI children differs from NH children in the social domains only; the levels of perceived physical appearance and global self-esteem do not differ from those of NH children. Improving language development and communication skills could help to build up higher levels of self-esteem regarding peers. Unfortunately, irrespective of their language and communication skills, HI children in special education show lower levels of self-esteem in the parental domain. The aim of this research was to create more awareness concerning this vulnerable group of children, resulting in increased attention and monitoring by professionals, in order to promote good mental health in each HI child.
